# A Capacitive 3-Axis MEMS Accelerometer for Medipost: A Portable System Dedicated to Monitoring Imbalance Disorders

**DOI:** 10.3390/s21103564

**Published:** 2021-05-20

**Authors:** Michał Szermer, Piotr Zając, Piotr Amrozik, Cezary Maj, Mariusz Jankowski, Grzegorz Jabłoński, Rafał Kiełbik, Jacek Nazdrowicz, Małgorzata Napieralska, Bartosz Sakowicz

**Affiliations:** Department of Microelectronics and Computer Science, Lodz University of Technology, 93-005 Lodz, Poland; piotr.zajac@p.lodz.pl (P.Z.); piotr.amrozik@p.lodz.pl (P.A.); cezary.maj.1@p.lodz.pl (C.M.); mariusz.jankowski@p.lodz.pl (M.J.); grzegorz.jablonski@p.lodz.pl (G.J.); rafal.kielbik@p.lodz.pl (R.K.); jacek.nazdrowicz@dokt.p.lodz.pl (J.N.); malgorzata.napieralska@p.lodz.pl (M.N.); bartosz.sakowicz@p.lodz.pl (B.S.)

**Keywords:** MEMS accelerometer, ASIC readout circuit, portable system, imbalance disorders

## Abstract

The constant development and miniaturization of MEMS sensors invariably provides new possibilities for their use in health-related and medical applications. The application of MEMS devices in posturographic systems allows faster diagnosis and significantly facilitates the work of medical staff. MEMS accelerometers constitute a vital part of such systems, particularly those intended for monitoring patients with imbalance disorders. The correct design of such sensors is crucial for gathering data about patient movement and ensuring the good overall performance of the entire system. This paper presents the design and measurements of a three-axis accelerometer dedicated for use in a device which tracks patient movement. Its main focus is the characterization of the sensor, comparing different designs and evaluating the impact of the packaging and readout circuit integration on sensor operation. Extensive testing and measurements confirm that the designed accelerometer works correctly and allows identifying the best design in terms of sensitivity/stability. Moreover, the response of the proposed sensor as a function of the applied acceleration demonstrates very good linearity only if the readout circuit is integrated in the same package as the MEMS sensor.

## 1. Introduction

Research into microelectromechanical systems (MEMS) is one of the most dynamically developing branches in microelectronics [1,2,3,4]. The devices can be found almost everywhere, from smartphones and the internet-of-things to clothes and many other applications. Most importantly, MEMS accelerometers are used in GPS-aided navigation systems [5,6,7], and military [8], automotive [9], aerospace [10] and medical devices [11,12,13,14]. Thanks to their miniature size they can also be easily used in healthcare applications. For example, two devices, SwayStar [15] and VertiGuard [16], can be used to monitor the gaits of patients by examining their movement. We propose a similar system developed in cooperation with the Medical University of Lodz [17,18] for patients with imbalance disorders. Medipost is a small, compact device which can be easily mounted on the back of the patient’s belt. It is supplied by a Li-Ion battery and can be used at home. Medipost communicates via Bluetooth with a smartphone [19], on which any received data is preliminarily processed by a dedicated application. Next, the data is transmitted to a PC located in a medical centre, where the patient’s movement can be monitored by a doctor and the proper treatment administered. Apart from monitoring movement, the system can also prevent serious injury by triggering a warning when the patient is about to fall.

On its basic level, Medipost uses MEMS inertial sensors and dedicated integrated readout circuits for measuring the acceleration and angular velocity of certain parts of the patient’s body, which allows precise evaluation of their movement and potential movement-related health issues.

However, the correct operation of the entire system requires the proper design of an acceleration sensor. Therefore, in this paper we present and characterize a custom accelerometer designed specifically for the Medipost device. The paper is organized as follows: Section 2 presents the operation principle and the design of the 3-axis accelerometer; Section 3 describes the simulations, as well as the measurement results of the sensor; and Section 4 presents our Conclusions.

## 2. MEMS Accelerometer Design and Manufacturing

### 2.1. Capacitive Accelerometer Operation Principle and Design

**Figure 1:**
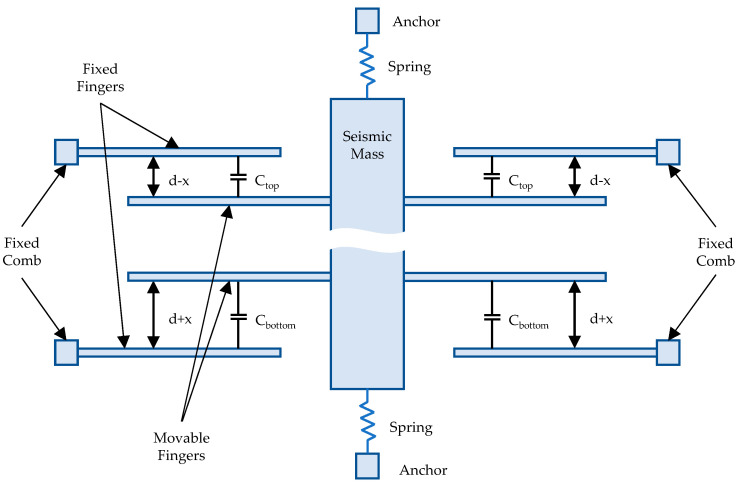
The operation principle of the capacitive accelerometer acting in the X and Y axes (not to scale).

We designed a 3-axis accelerometer, consisting of three independent capacitive single-axis accelerometers, which operates by measuring changes in capacitance caused by applied acceleration. The accelerometer itself consists of a seismic mass hanging on springs over a substrate [20]. Combs are attached to the mass, forming a movable part of the sensor. The same number of parallel combs is in turn connected to a frame which forms a fixed part of the sensor, thus forming two capacitors with movable and fixed plates on both sides of the seismic mass. The concept of a single-axis accelerometer is shown in Figure 1. In the sensor described in this paper, two accelerometers have been designed according to this principle: one operating in the X-axis and another in the Y-axis.

As shown in Figure 1, the design consists of two capacitors: C*_top_* and C*_bottom_*. When accelerated, the mass moves, and one of the capacitances increases while the other decreases due to the displacement of the fingers. The difference between these capacitances can be measured, thus the magnitude of acceleration can be derived. The value of the capacitances C*_top_* and C*_bottom_* after acceleration is applied is given by:

(1) $$C_{top}=\varepsilon nS\left(\frac{1}{d-x}\right),\;\;C_{bottom}=\varepsilon nS\left(\frac{1}{d+x}\right)$$

where *S* is the surface of a single finger, *d* is the distance between fingers, *x* is the displacement caused by acceleration, *n* is the number of fingers which form a single capacitor plate and *ε* is the electrical permittivity of the material between the fingers.

As the principle of operation of such a sensor is well described in literature [21], we have only presented the most important equations necessary to understand the accelerometer’s behaviour; these are given in Appendix A of this paper.

**Figure 2:**
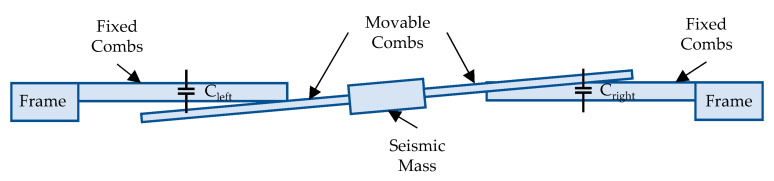
The operation principle of the capacitive accelerometer in the Z axis (rotation not to scale).

Although we have also designed a Z-axis accelerometer, it has to be emphasized that it has a slightly different design to the X- and Y-axis accelerometers described above [22]. While it is also constructed with two combs, which form C_left_ and C_right_ capacitors (Figure 2), the design of the seismic mass is asymmetric to obtain a capacitance change upon acceleration. In this design, the mass with movable fingers rotates in the XZ plane, which is presented in Figure 2.

Two types of fingers are used in this design: normal fingers, as fixed combs, and thinned fingers (with reduced height), as movable combs. When acceleration is applied, only one capacitance changes significantly, because its corresponding comb moves out from the overlapping area, thus allowing the direction of the applied acceleration to be determined. However, the disadvantage of such an approach is that it demonstrates only half the sensitivity with respect to X- and Y-axis accelerometers. This asymmetric comb-drive design was necessary because it is impossible to create an electric connection on the substrate: the chosen technology does not allow the deposition of a metal layer on the substrate in a cavity.

**Figure 3:**
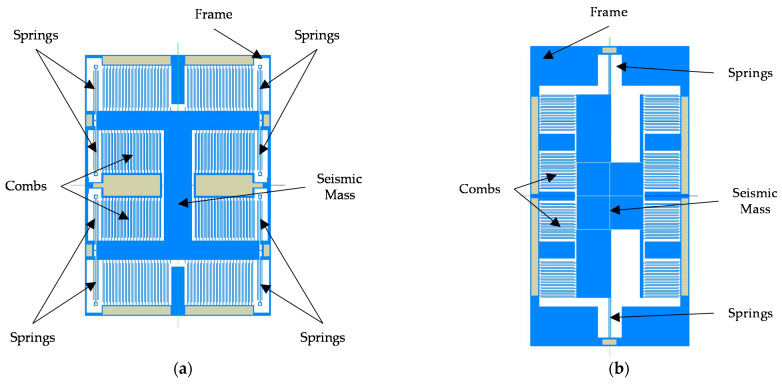
Models of the designed accelerometers: (**a**) *X*-axis accelerometer; (**b**) *Z*-axis accelerometer.

The final version of the 3-axis accelerometer is composed of two accelerometers based on a standard comb-drive design (Figure 3a) and one accelerometer based on an asymmetric comb-drive design (Figure 3b). The first two allow the acceleration to be measured in the X and Y axes and the third in the Z axis. Additionally, as the first manufacturing run of the device was a trial, it was decided to test two different designs for X- and Y-axis accelerometers. The X-axis device was made 1.5 times larger than the Y-axis one: consequently, it has noticeably higher sensitivity but at the cost of a longer response time.

### 2.2. Dedicated Readout Circuit Design

The presented accelerometer requires ReadOut Integrated Circuit (ROIC) [23,24] in order to process signals from the sensor. We designed three different independent structures for each axis with quite small sensitivities (e.g., 0.31 fF/g for Z axis) and differential outputs. Therefore, we designed three independent readout channels optimized for each structure. This approach has the following advantages:

- Each structure of the MEMS sensor can have its own dedicated readout circuit whose parameters are tailored to this particular structure.
- It allows us to align the bonding pads of the readout circuit with the pads of the sensor. It equalizes the bonding wire lengths and creates a regular, symmetrical connection structure between both dies, minimizing the influence of parasitics.
- Each of the three readout channels is digitally configurable, i.e., the parameters of the channel, such as: gain, reference voltages, mismatch compensation, switching frequency, etc., can be easily adapted to application requirements.

The ROIC converts the signal from the MEMS sensor (capacitance difference) into voltage to amplify it and to digitize it using an integrated analog-to-digital converter (ADC). It operates in a differential mode and uses switched-capacitor circuits. The digitized signal can be sent via SPI interface to a separate microcontroller, which can then transmit the data wirelessly using Wi-Fi or Bluetooth. More detailed information on the designed ROIC has been given previously [20].

### 2.3. Manufacturing of the Accelerometer

**Figure 4:**
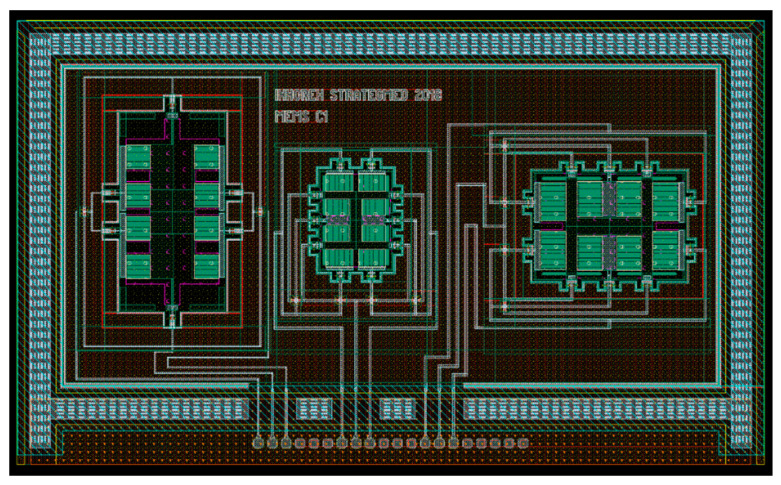
3-axis MEMS accelerometer layout. (**right**) X-axis accelerometer, (**centre**) Y-axis accelerometer, (**left**) Z-axis accelerometer.

**Figure 5:**
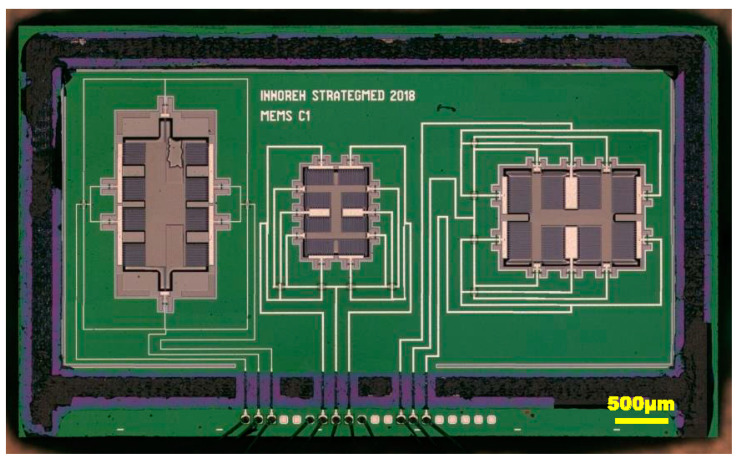
3-axis MEMS accelerometer photography. (**right**) X-axis accelerometer, (**centre**) Y-axis accelerometer, (**left**) Z-axis accelerometer.

**Figure 6:**
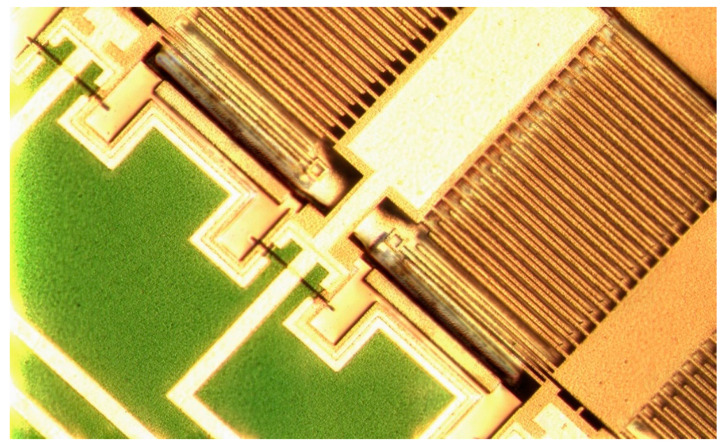
X-axis accelerometer photography.

The sensor was designed and manufactured in XFAB MEMS 3D capacitive technology [25]. Its layout and photography are presented in Figure 4 and Figure 5, respectively. Three independent sensors are used, one for each axis. This approach to the design is simpler in comparison to one with a single seismic mass for all axes. It also reduces the cross-axis sensitivity. Moreover, it allows each structure to be optimized individually. One of the disadvantages is the larger size of the accelerometer. The designs of our X-axis and Y-axis sensors follow the classic approach used in capacitive parallel-plate MEMS accelerometers (Figure 6). Meanwhile, the Z-axis sensor has a slightly different design; please refer to [22] for details. Since the sensor structure is differential, special consideration is taken to ensure that the lengths of all signal paths are the same to minimize the impact of parasitic capacitances on the sensor output. In addition, all structures are integrated in an enclosed package to protect them from mechanical damage or external interference.

**Figure 7:**
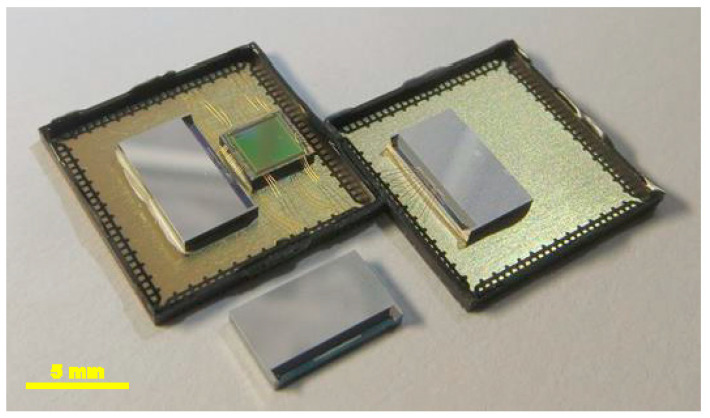
All variants of the MEMS structure packaging: naked die with MEMS sensor only (**bottom**), MEMS sensor-only structure in a QFN-100 package (**right**) and the MEMS sensor bonded with the ROIC in the same QFN-100 enclosure (**left**).

Although both the sensor and the ROIC were integrated in the same package to ensure the best performance, both were also manufactured in their own separate packages to allow them to be studied separately (Figure 7). In addition, we also received an unpackaged version of the sensor chip (a naked die) to allow measurements to be conducted on a probe station.

**Figure 8:**
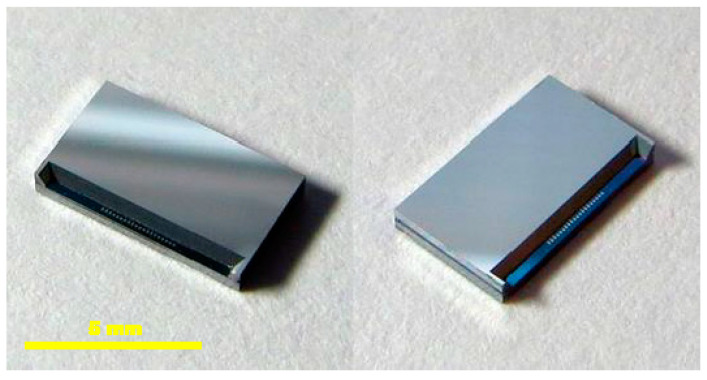
Close-up views of the naked sensor dies.

All variants of the accelerometer packaging are given in Figure 7: the naked die with MEMS sensor only (bottom), the MEMS sensor-only structure in a QFN-100 package (right), and the MEMS sensor bonded with the corresponding readout circuit in the same QFN-100 enclosure (left). Figure 8 depicts a close-up view of the naked sensor dies.

The present study focuses mostly on the MEMS device. The main goal of the paper is not only to characterize the designed accelerometer, but also to verify the impact of parasitic capacitances induced by the pads and packaging on sensor performance. It should be emphasized that the measured capacitances are very small and output signals can be quite easily disrupted by large parasitics.

## 3. Simulation Results and Measurements

**Figure 9:**
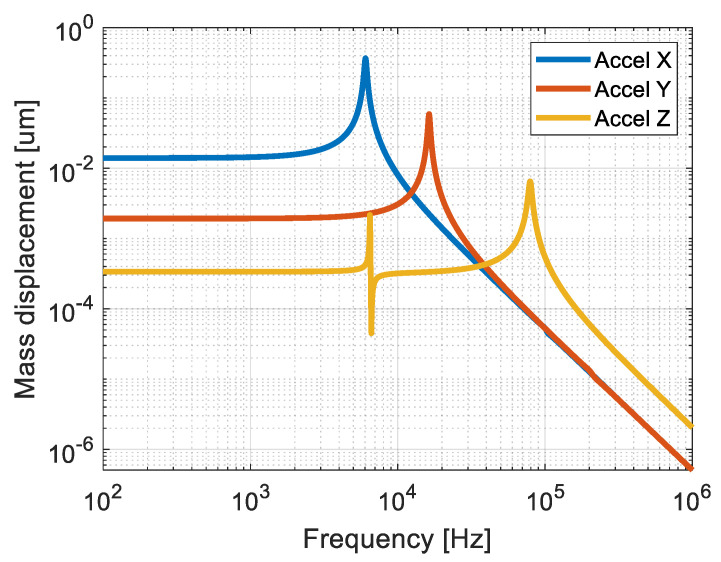
Frequency response of the accelerometer.

The MEMS accelerometers were designed in Coventor MEMS+ software [26]. The software uses fundamental MEMS-specific building blocks (combs, anchors, etc.) which are combined to create a complete design. The simulations of such models are up to 100 times faster than those performed in Finite Element Analysis tools, according to the Coventor MEMS+ developer [27]. Additionally, the Coventor model of the device can be easily exported to Cadence Virtuoso software [28], in which it can be simulated together with the corresponding readout circuit. Table 1 lists the sensitivities and first eigenfrequencies found for each sensor. In Figure 9, the frequency response of the sensor is presented. As expected, the most sensitive structure (*X*-axis accelerometer) has the lowest eigenfrequency (Equation (A2) in Appendix A).

### 3.1. Accelerometer Capacitance Measurements Using Impedance Analyzer

**Figure 10:**
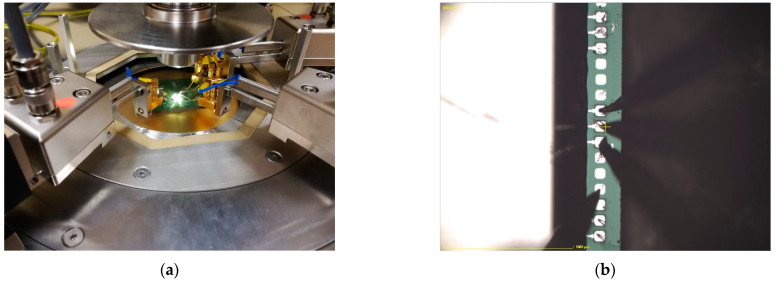
The measurements of the naked die performed on the probe station. General view of the device on the probe station (**a**). Zoom of the probes placed on the chip’s pads (**b**).

In order to characterize the device, static measurements were performed using Summit 11000/12000 Probe Station (Cascade Microtech, Beaverton, OR, USA). The naked die was placed in the probe station and probes were placed on chip’s pads (see Figure 10). A Keysight E4990A Impedance Analyzer was then used to measure the capacitance of each accelerometer. The obtained results are shown in Table 2, which also provides simulated values obtained from Coventor MEMS+ for comparison purposes. Good agreement was observed between measurements and simulation results.

As it is impossible to apply the acceleration to the device placed in the probe station, another method had to be used to measure the responses of the accelerometers to applied loads. The seismic mass was displaced by applying a voltage to one of the capacitances; the induced electrostatic force causes a capacitance change, similar to that obtained by acceleration [29].

**Figure 11:**
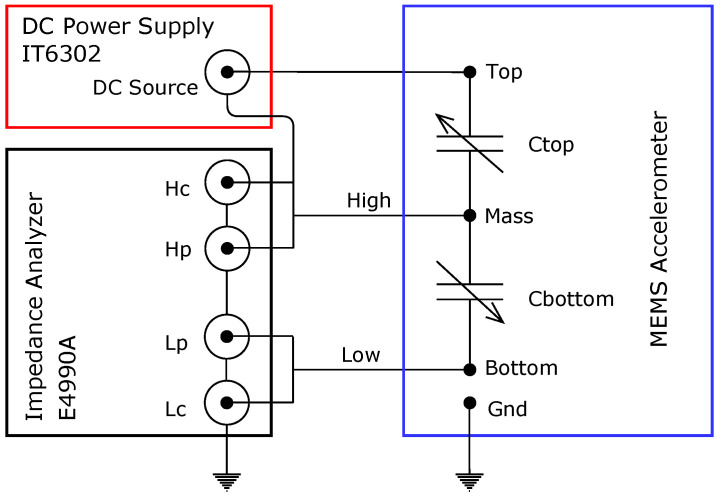
Measurement setup for the electrostatic actuation of the MEMS accelerometer.

The measurement setup is shown in Figure 11. A DC voltage was applied across one capacitance C_top_ while the second capacitance C_bottom_ was measured using the impedance analyser. Following this, the roles of C_top_ and C_botttom_ were swapped and the measurements were repeated. It was found that the results of such reversed measurements were almost identical to the original ones.

This method allows the capacitances to be measured as a function of the applied DC voltage in the *X*- and *Y*-axis accelerometers. This approach is not viable in the case of the *Z*-axis accelerometer due to its design.

**Figure 12:**
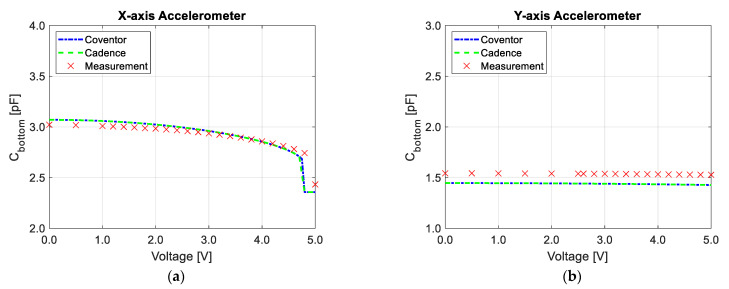
The value of C_bottom_ as a function of applied voltage: (**a**) X-axis accelerometer; (**b**) Y-axis accelerometer.

The measurements and simulations performed in Coventor MEMS+ and Cadence are compared in Figure 12. In the larger structure (*X*-axis sensor), a pull-in effect can be observed when a voltage of around 4.7 V is applied between the plates. It was verified that this effect is reversible and does not damage the sensor. In the case of the Y-axis sensor (Figure 12b) the pull-in effect is not observed in the range of applied voltage, because the structure is more rigid. The measurements correlate very well with the simulations.

### 3.2. Accelerometer Capacitance Measurements with External Readout Circuit

**Figure 13:**
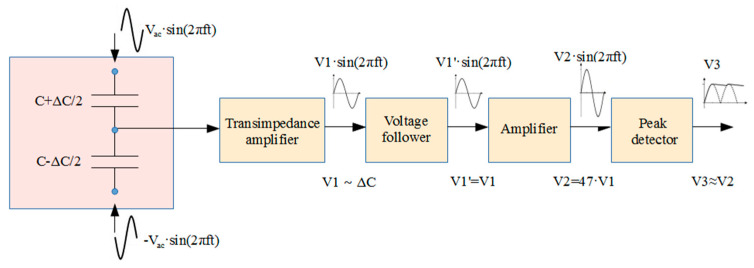
Operating principle of the designed external readout circuit.

The sensitivity of the MEMS device is around 13.15 fF/g in the X-axis and around 0.85 fF/g for the Y-axis. Measuring such small capacitance changes using a Keysight E4990A impedance analyser can be a challenge due to its limited resolution. Therefore, an external readout circuit (ERC) was designed and built. It uses the standard approach of applying a differential sinusoidal signal to the top and bottom electrodes of the capacitive sensor and reading the output signal at the middle node (Figure 13). The middle node signal is converted to the voltage V1, whose amplitude should be proportional to the capacitance change ΔC, using a transimpedance amplifier. High impedance is ensured at the output of the transimpedance amplifier using a voltage follower. Subsequently, a 47× gain is applied by the inverting amplifier. Note that the output signal is a high-frequency signal; therefore, in the final step, these high frequencies are removed using an active peak detector. As a result, the amplitude of the output voltage V3 is proportional to ΔC and can be measured using an oscilloscope.

The choice of the appropriate voltage V_ac_ and the frequency *f* depends on the parameters of the measured MEMS device. In our case, it was determined that the use of V_ac_ between 1–3 volts and the frequency between 200–500 kHz produces the best results.

The ERC was used with the probe station (using a similar setup as in Section 3.1). Two complementary input signals were applied by a signal generator. Its outputs were synchronized to provide two sinusoids with the same amplitude and frequency but inverted phases. The signals were applied through probes to the top and bottom MEMS electrodes. The output was read by the probe from the middle electrode and used as an input to the ERC circuit. To obtain a capacitance change, a variable DC offset voltage was applied together with the input signals, generating an electrostatic force between the accelerometer’s plates.

The initial measurements were performed on the probe station for the naked die. Following this, another set of measurements based on the same method was run for the packaged chip, with the probes placed on the external pins of the chip. Finally, the same measurements were repeated once more, this time with the MEMS sensor mounted on the PCB and probes injected into the PCB pads.

**Figure 14:**
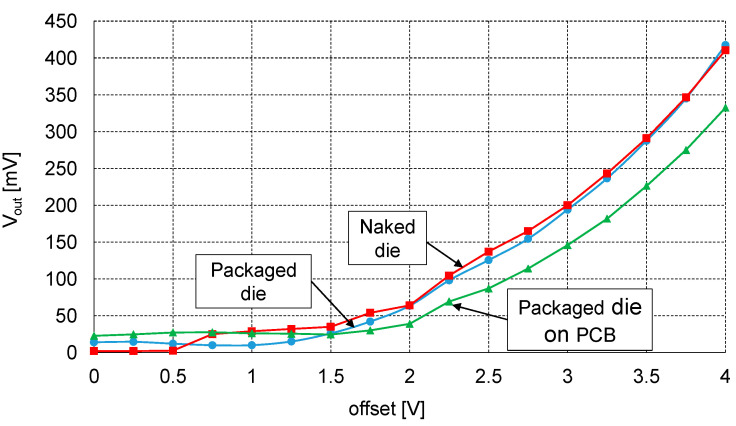
Comparison of results for three sets of measurements (for the naked die, for the packaged die and for the packaged die mounted on the PCB).

Figure 14 shows the obtained dependence of the amplitude of the output voltage V2 as a function of the applied voltage offset. Several interesting conclusions can be drawn by analysing these results. For voltages below 1.5 V, the output signal was mostly noise; however, a clear sinusoid was visible at the ERC output after exceeding 1.5 V. The similarity between the curves for the naked and packaged die indicates that adding the chip package (inserting gold wires and chip pins to the signal path) does not produce any noticeable difference in the results. Thus, one may assume that even if some parasitic package capacitances are added, they are equal on both signal lines and therefore do not influence the difference in capacitance. On the contrary, reading the sensor output from the PCB pads produces very different results: for example, for the highest voltage of 4 V, the measured output voltage amplitude was about 20% lower than the one obtained for the naked die. The reason is that the output amplitude depends on the difference between C_top_ and C_bottom_. Consequently, when the PCB pads and connections introduced additional parasitic capacitances in parallel to those two capacitances, the difference between C_top_ and C_bottom_ decreased, causing an error in the readout.

### 3.3. Gravitional Acceleration Measurements

**Figure 15:**
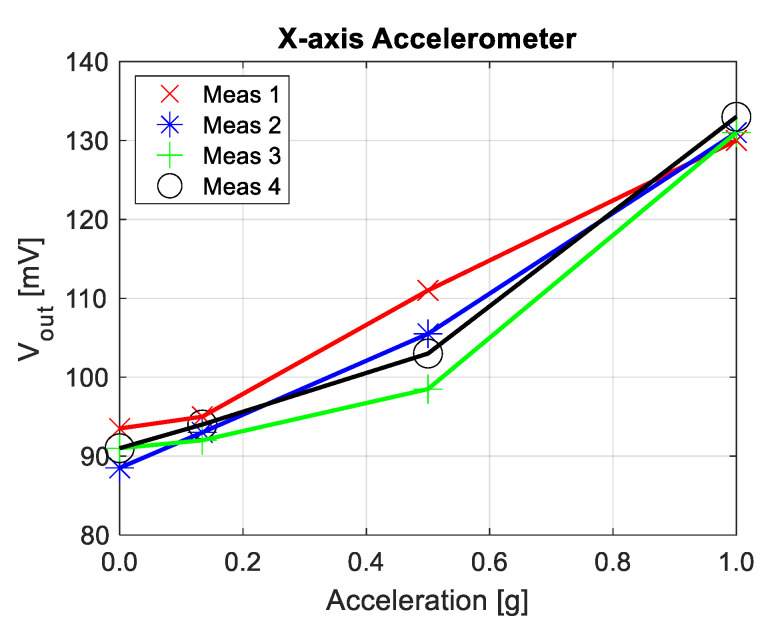
The measured output voltage of the ERC as a function of acceleration. The measured device was positioned at four angles (0, 30, 60 and 90 degrees). The same measurements were repeated four times.

As a final test, the MEMS chip on the PCB was mounted on a movable lever to study the response of the sensor to gravitational acceleration. As in previous measurements, an ERC was used to read the voltage output signal proportional to the measured capacitance change. By moving the lever and varying the angle at which the MEMS sensor was positioned, we were able to determine the dependence of the output voltage on the acceleration. Four sets of measurements were performed for four different angles (see Figure 15). It can be seen that, especially in the middle of the acceleration range, substantially different values were measured for the same angle (0.5 g) and the dependence is not exactly linear, as could be expected. However, it should be noted that the measurements were very susceptible to any external interference. In particular, the signal corresponding to the middle node of the accelerometer was very sensitive to any change in its position with respect to other connections. Even after isolating these connections, the output signal was quite noisy and unstable, which made these measurements challenging.

The results obtained in this section confirm that, to ensure the stability of the measurements, the readout circuit for capacitive MEMS accelerometers should always be integrated in the same package as the sensor. The capacitance change of such accelerometers is very small, in the range of femtofarads per g, and therefore the signal at the middle node of such differential sensors is also quite small and easily disrupted by noise, external interference, or the impact of parasitics.

**Figure 16:**
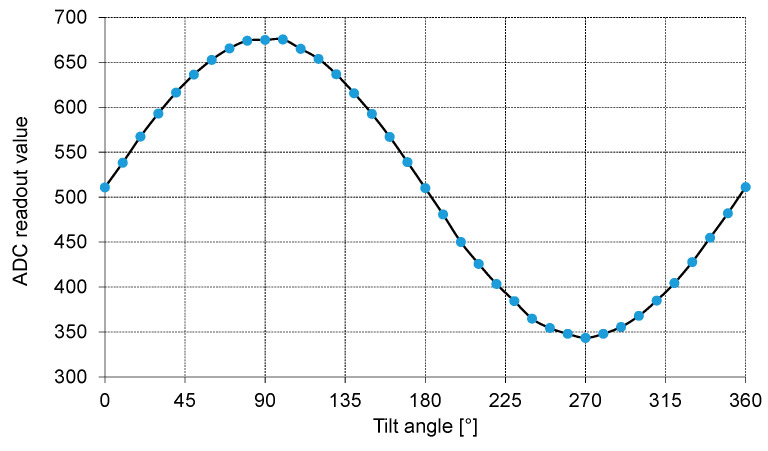
The output of the ROIC as a function of the tilt angle.

**Figure 17:**
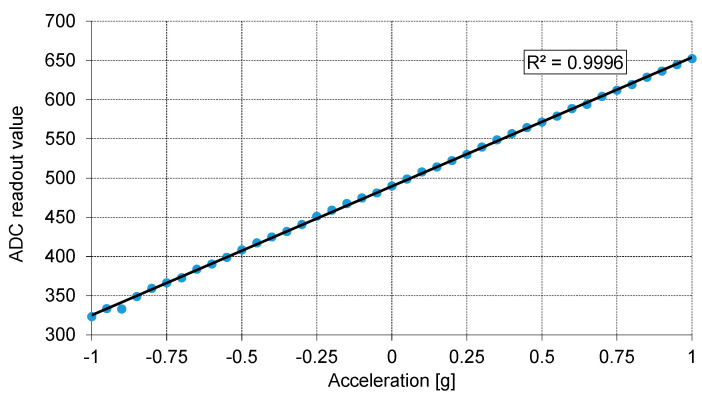
The output of the ROIC of the X-axis sensor as a function of gravitational acceleration.

**Figure 18:**
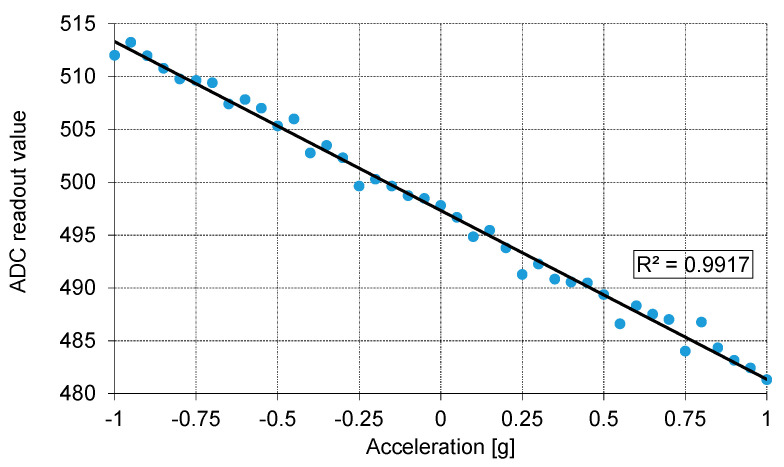
The output of the ROIC of the Y-axis sensor as a function of gravitational acceleration.

To demonstrate this, the results obtained for the X-axis and Y-axis accelerometers with an integrated readout circuit (ROIC, Section 2.2) are given in Figure 16, Figure 17 and Figure 18. The measurements appear to be completely stable and the output has an almost perfectly linear dependence on the acceleration. The selected tests are straightforward, and their results are easy to interpret. In Figure 16, ROIC output was measured as a function of the tilt angle. The tilting device used during the conducted procedure was calibrated both in degrees and fractions of gravitational acceleration (g). The output of the ROIC was measured at 10-degree intervals and the output vs. g fraction was taken every 0.05 g. To minimize any unwanted influence of issues related to the test setup, the ADC output was averaged to produce individual data points for each tested tilt angle and g fraction.

The presented data were read from the 10-bit ADC built into the ROIC with the MEMS sensor at its input. A built-in mismatch compensation circuit was used to shift the output offset to ensure that zero acceleration produced an output level roughly in the middle of the entire range (0–1023). For the X-axis (Figure 16 and Figure 17) the ADC range corresponds to the measurement range of about ±2.85 g, to comply with requirements of the methodology used in the Medipost device. It can be observed that the output capacitance of the MEMS sensor has a quite regular sinusoidal dependence on the tilt angle, and a clearly linear dependence on *g* fraction (R^2^ = 0.9996 for X axis). Slightly worse results in terms of linearity (R^2^ = 0.9917) were obtained for the Y-axis accelerometer (Figure 18). Thus, both the MEMS sensor and the corresponding ROIC integrated in the same chip package appear to work correctly and may be implemented in the Medipost device.

### 3.4. Comparison of the Three-axis Multiple Sesmic Mass Accelerometers

To summarize our investigations, we compare our design with various accelerometers whose parameters have been published in literature [30]. We only considered sensors with three separate one-axis structures and multiple seismic masses (three seismic masses, one per one-axis structure). The most important features of the selected accelerometers are collected in Table 3.

The range of our accelerometer results from the requirements of its medical application. Following the guidelines from medical staff, it was established that a range of ±2.5 g would be enough to adequately track the movement of patients. The device size is quite typical for this type of accelerometer. The sensitivity (in fF/g) on the other hand is low, as we decided to design quite rigid structures; this allowed higher linearity and avoided damaging the sensor in case of excessive accelerations (for example, during a patient’s fall). Still, the sensitivity of the ROIC output (in mV/g) is satisfactory. The obtained linearity is very good; however, we have to emphasize that it was so far only measured in the range of ±1 g (a wider range of measurements is planned for the future).

## 4. Conclusions

This paper presents the design and test results of a 3-axis accelerometer used in a portable system for monitoring imbalance disorders. The device was constructed by combining three independent single-axis accelerometers. As the first manufacturing run was only a trial, with a second, final run being planned, the accelerometers for the X- and Y-axes differed in size to explore the design space. Our findings indicate that the larger structure, i.e., the X-axis, is better suited for the application, as it provides higher sensitivity and its lower bandwidth is acceptable.

Initially, we performed an extensive characterization of the MEMS sensor without the ROIC. First, the measurements of the sensor capacitance as a function of applied voltage showed good agreement with software simulations; even the pull-in effect was correctly predicted. Following this, we tested the X-axis accelerometer in three configurations: as a naked die, a packaged die, and as a package mounted on the PCB. It was discovered that adding a package does not introduce any noticeable changes in the sensor output; however, a visible difference in the results was observed for the sensor mounted on the PCB compared to the two previously analysed cases. Thus, it may be concluded that when splitting the system into separate MEMS and ROIC packages, the weak capacitive MEMS sensor signal is affected by the parasitics of PCB connections and pads, causing a significant error in the output value (up to 20% in the analysed case).

Our findings indicate that to obtain the best performance, the capacitive accelerometers intended for the MEMS should be designed with an integrated ROIC. This was further proved by testing X-axis and Y-axis accelerometers with the ROIC integrated in the same package: a very stable output and reproducible measurements were obtained, and a very good linearity was observed in the analysed ±1 g range.

## 5. Patents

Two relevant patents were granted by The Patent Office of the Republic of Poland:

- Three-axis acceleration sensor layout in XMB10 MEMS technology (INNOREH MEMS C1), Authors: Szermer, M., Maj, C., Zając, P., Nazdrowicz, J. Application Number: S.0071, Application Date: 03.02.2021
- Readout integrated circuits layout for a three-axis acceleration sensor in CMOS 180 nm technology (INNOREH ASIC C1), Authors: Amrozik, P., Kiełbik, R., Zając, P., Jankowski, M., Jabłoński, G. Application Number: S.0072, Application Date: 3 February 2021

## Figures and Tables

**Table 1:** List of parameters of the X-, Y- and Z-axis sensors.

Accelerometer Type	Resonance Frequency [kHz]	Sensitivity [fF/g]	Max. Measurable/min. Detectable (Ideal, i.e., no Noise) Acceleration [mg]	Resolution at Operating Bandwidth (10 Hz) [mg]
*X* axis	6.043	13.15	±2.85/5.57	12.8
*Y* axis	16.321	0.85	±2.94/5.74	140
*Z* axis	6.484	0.31	±4.03/7.87	–

**Table 2:** Measurement and simulation results of the nominal capacitance of the *X*-, *Y*- and *Z*-axis accelerometer.

Accelerometer	C_0_ [pF]	
	Simulations	Measurements
X-axis	3.070	2.933
Y-axis	1.446	1.449
Z-axis	1.445	1.397

**Table 3:** Parameters of three-axis multiple seismic mass accelerometers.

Ref.	Year	Device Size [mm × mm]	Range X, Y, Z [±g]	Sensitivity X, Y, Z	Nonlinearity X, Y, Z
[31]	1999	4 × 4			
			1.9	0.4 fF/bit	–
		
[32]	1999	5 × 5		25 fF/g	
			–	25 fF/g	–
				100 fF/g	
[33]	2005	7 × 9		6.8 pF/g	
			1	6.8 pF/g	–
				2.9 pF/g	
[34]	2013	1.57 × 1.73		105 mV/g	1%
			0.01 ÷ 2	127 mV/g	0.5%
				58 mV/g	2.4%
[35]	2015	12 × 7	10		0.34%
			10	–	0.28%
			+12, –7		0.41%
This work	2018	3.95 × 6.55	2.85	13.15 fF/g, 701 mV/g	0.40% (R^2^ = 0.9996) for ±1 g range
			2.94	0.85 fF/g, 680 mV/g	0.83% (R^2^ = 0.9917) for ±1 g range
			4.03	0.31 fF/g, 496 mV/g	–

## Data Availability

Not available.

## Appendix A

The operation principle of the presented accelerometer can be described by the following equation:

(A1) $$m\frac{d^{2}x\left(t\right)}{dt^{2}}+kx\left(t\right)=ma_{x}\left(t\right)$$

where *m* is the mass of the movable part of the accelerometer, *k* is a suspension stiffness, *x*(*t*) is a position of the seismic mass along the X axis as a function of time and *a_x_*(*t*) is the value of the component of the acceleration vector parallel to the X axis. On the basis of Equation (A1), the eigenfrequencies can be obtained by the following formula:

(A2) $$f_{0}=\frac{1}{2\pi }\sqrt{\frac{k}{m}}$$

The above equations are valid when the sensor operates in a vacuum. The air surrounding the sensor has an influence on its movement and it is necessary to add an additional term responsible for damping to the Equation (A1):

(A3) $$m\frac{d^{2}x\left(t\right)}{dt^{2}}+b\frac{dx\left(t\right)}{dt}+kx\left(t\right)=ma_{x}\left(t\right)$$

where *b* is the damping coefficient.

According to this equation, we can see that damping force is proportional to the seismic mass velocity. Although this is true if the sensor is located alone in the air (i.e., without the package), this is not the case if it is placed in a small package with very small distance from the frame: the air in the gap compresses or expands during movement, which is a non-linear phenomenon and has little to do with the most-common viscous damping effects. In the literature, this phenomenon is known as a squeeze-film damping effect [36,37]. This effect has a small influence on the movement of the seismic masses in sensors for the X- and Y-axis, where fingers are used to create the capacitor plates. In these sensors the bandwidth is much wider than in the sensors in the Z-axis, where the damping is much higher.

If we add the following components to the Equation (A3):

(A4) $$\omega_{0}^{2}=\frac{k}{m}\;\mathrm{and}\;\beta =\frac{b}{2m}$$

We obtain the following equations:

(A5) $$\frac{d^{2}x\left(t\right)}{dt^{2}}+\frac{b}{m}\frac{dx\left(t\right)}{dt}+\frac{k}{m}x\left(t\right)=a_{x}\left(t\right)$$

(A6) $$\ddot{x}+2\beta \dot{x}+\omega_{0}^{2}x=a_{x}\left(t\right)$$

where $\omega_{0}^{2}$ is a pulsation of undamped vibrations and *β* is the normalized damping factor.

Fourier transformation of Equation (A6) results in the following equation:

(A7) $$\omega^{2}x\left(j\omega \right)+j2\beta \omega x\left(j\omega \right)+\omega_{0}^{2}x\left(j\omega \right)=a_{x}\left(j\omega \right)$$

Next, we can calculate the frequency response of the model:

(A8) $$T\left(j\omega \right)=\frac{x\left(j\omega \right)}{a_{x}\left(j\omega \right)}=\frac{1}{\omega_{0}^{2}+\omega^{2}+j2\beta \omega }$$

It is possible to obtain the amplitude and phase characteristics from Equation (A8) by calculating the modulus and argument of the above expression, respectively.

If *β* < *ω*_0_, we receive the resonant curve with the following resonance frequency:

(A9) $$f_{r}=\frac{\sqrt{\omega_{0}^{2}-\beta^{2}}}{2\pi }$$

Hence, the natural frequency decreases as damping increases. If the damping is sufficiently strong, no resonance will occur at all.
